# UPLC-Q/TOF-MS-Based Serum Metabolomics Reveals Hypoglycemic Effects of *Rehmannia glutinosa*, *Coptis chinensis* and Their Combination on High-Fat-Diet-Induced Diabetes in KK-Ay Mice

**DOI:** 10.3390/ijms19123984

**Published:** 2018-12-11

**Authors:** Zhenxian Qin, Wei Wang, Dengqun Liao, Xiaoying Wu, Xian’en Li

**Affiliations:** Institute of Medicinal Plant Development, Chinese Academy of Medical Sciences & Peking Union Medical College, Beijing 100193, China; qinzhenxian@126.com (Z.Q.); wangwei9062@163.com (W.W.); dqliao@implad.ac.cn (D.L.); wuxiaoying_cpu@163.com (X.W.)

**Keywords:** *Rehmannia glutinosa*, *Coptis chinensis*, hypoglycemic effect, high-fat diet, diabetes, metabolomics, UPLC-Q/TOF-MS

## Abstract

Diabetes is a worldwide severe health issue which causes various complications. This study aimed to evaluate the hypoglycemic effects of *Rehmannia glutinosa* (RG), *Coptis chinensis* (CC) alone and their combination on high-fat-diet-induced diabetes in mice via biochemical assays and UPLC-Q/TOF-MS-based serum metabolomic analysis. Diabetic KK-Ay mice were induced by high-fat diet and treated for eight weeks, separately with RG, CC and their combination and the positive control drug metformin. Administration of RG and CC alone, and their combination could decrease the fasting blood glucose level, ameliorate the tolerance of glucose, and recover the levels of total cholesterol (TC), triglycerides (TG), high-density lipoprotein cholesterol (HDL-C), and low-density lipoprotein cholesterol (LDL-C) in sera of diabetic mice. Orthogonal partial least squares discriminant analysis (OPLS-DA) on serum metabolomes revealed that 79 ESI^+^ and 76 ESI^−^ metabolites were changed by diabetes mellitus (DM) compared to the normal control. Heatmaps on these diabetes-related metabolites showed that CC and RG/CC were clustered closer with the normal control, indicating that they had the better antidiabetic effects at the metabolite level. Fifteen of the differential metabolites in DM serum were annotated and their related metabolic pathways were lipid metabolism. These data suggested that RG and CC alone and in combination treatment had the antidiabetic activity in lowering glycemia and improving lipid metabolism. UPLC-Q/TOF-MS-based metabolomics shed light on the differential metabolite effects of RG and CC in DM treatment. However, it should be noted that some differential metabolites were possibly generated or not detected due to our groupwise run order, which possibly contributed to or covered the group difference in our experiment. They need to be further discriminated in the future work.

## 1. Introduction

Diabetes mellitus (DM) is a complicated and systemic metabolic disease caused by the interplay of multiple factors including genetic predisposition, behavioral and environmental risk factors [1]. It is characterized mainly by hyperglycemia and dyslipidemia resulting from the deficient insulin secretion and/or insulin resistance. The persistent elevation of blood glucose in diabetic patients caused many common long-term and acute complications including coronary heart disease, stroke, diabetic retinopathy, ulceration, dementia, and nephropathy [2,3,4,5]. Due to the unhealthy diet and less routine physical exercise, DM is rapidly increasing worldwide and has become the third largest common chronic disease following the cardiovascular disease and cancer. The International Diabetes Federation (IDF) (https://www.idf.org) estimated that more than 425 million adults suffered DM in 2017 and this number would reach 629 million in 2045. Fortunately, some antidiabetic medicines such as biguanides, sulfonylureas, injectable insulins and thiazolidinedione are available in the current pharmaceutical market. However, these medicines especially in long-term use displayed many potential adverse effects such as weight gain, stomach upset, lactic acidosis, low blood sugar, anemia risk, skin rash and itching [6,7,8]. Therefore, it is significant to develop other alternative DM therapeutic drugs and natural herbal remedies with high efficiency and low toxicity [9,10]. 

According to the latest statistics, more than 1000 plants with medicinal properties are being used to treat DM worldwide [11]. Many Chinese herbal formulae consist of two or more herbs, which have multi- bioactive compounds that could exert synergistic therapeutic efficacies with less toxicity and fewer side effects [12,13,14]. Huanglian bolus was first recorded in “Qian-Jin-Yao-Fang” by Sun Si-miao in Tang Dynasty. It consists of the roots of *Coptis chinensis* (abbreviated as CC) Franch. and *Rehmannia glutinosa* (abbreviated as RG) Libosch. and has been used in ancient China for Xiao-ke symptom (a synonym of current DM name in traditional Chinese medicine). Although RG and CC alone had good therapeutic effects on diabetes and its complications [15,16,17,18], CC served as a sovereign drug while RG as a minister drug [19]. Rodent model-based experiments showed that their combined use had better pharmacological and therapeutic effects on diabetes than its single herb [19,20,21,22]. The underlying antidiabetic molecular mechanisms of Huanglian bolus and its separated herbal components especially at the metabolic level in DM organisms are less studied. Unavoidably, it was very challenging to understand the comprehensive mechanisms of action of compound traditional Chinese medicine, based on its multi-bioactive components acting on multi-targets [23]. Therefore, it is of great importance to explore the therapeutic mechanisms of Huanglian bolus on DM using an applicable and high throughout strategy.

Emerging metabolomics appeared an ideal tool to provide new insights into understanding the therapeutic effects and mechanisms of multi-biochemical component medicines [24,25]. This technology has the capability not only to provide the global metabolic changes caused by biochemical effects of toxins and drugs in biological samples such as plasma, urine, and tissues, but also to reveal the multiple related biochemical pathways of altered metabolites and pathophysiological states [26]. Like nuclear magnetic resonance (NMR) spectroscopy and GC-MS, LC-MS became one of most high throughput technologies in metabolomic research and has gradually been used to evaluate the therapeutic effects of traditional Chinese medicine (TCM) formula [24,25,26,27], due to its high sensitivity, great reproducibility, and wide metabolome coverage.

In this study, the untargeted UPLC-Q/TOF-MS-based metabolomics was conducted to investigate the potential modulating effects of RG and CC alone and their combination on the metabolites in sera of diabetic KK-Ay mice and further compare their pharmacological differences. Metabolites in response to the treatment of RG and CC and RG/CC against DM were identified and the underlying metabolic pathways were predicted.

## 2. Results

### 2.1. RG, CC, and Their Combination Decreased Fasting Blood Glucose Levels in HFD-Induced Diabetic Mice

**Figure 1:**
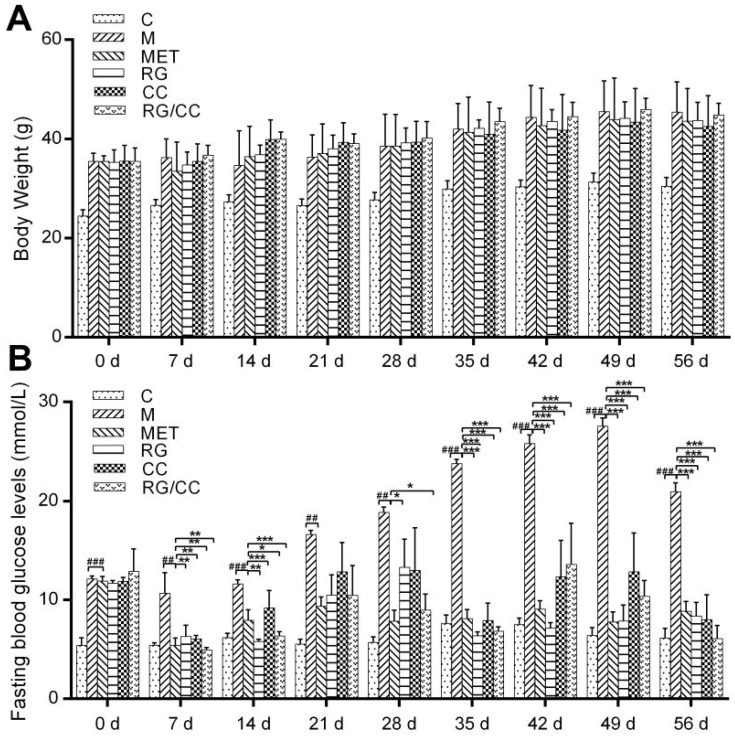
Changes of body weight (**A**) and fasting blood glucose levels (**B**) in different treatments of mice over 56 days of the experiment. ^##^ *p* < 0.01, and ^###^ *p* < 0.001 meant that the FBG value of the indicated group showed significant difference compared with the normal control group (C); * *p* < 0.05, ** *p* < 0.01, and *** *p* < 0.001 meant the FBG value of the indicated group was significantly different from the diabetic model control group (M). MET, RG and CC, RG/CC here and the below indicated diabetic mice groups administered separately with metformin (MET), decoctions of *Rehmannia glutinosa* (RG), *Coptis chinensis* (CC) and their combination (RG/CC).

The body weight and fasting blood glucose (FBG) levels of all experimental groups were measured every seven days during 56 days of medication treatment (Figure 1A,B). Body weight loss is one of three typical symptoms for diabetic patients. Although it was observed that water extract of raw RG radix could greatly increase the weight of high-fat diet (HFD) and streptozotocin (STZ)-induced diabetic mice [28]; however, the body weight of all the groups in our study increased with the experimental time and showed no significant difference among the medication groups and the model control group (Figure 1A). This result was also found in the study [29] using KK-Ay mice with the spontaneous obesity diabetic symptom. The normal control mice fed normal diet had the steady FBG levels during the experiment, ranging on average from 5.40 ± 0.76 mmol/L (at Week 0) to 7.61 ± 0.86 mmol/L (at Week 5). After KK-Ay mice were fed HFD for two weeks, the FBG levels in HFD-fed KK-Ay mice increased to around 12 mmol/L and significantly higher than that of the normal control mice fed normal diet (5.4 mmol/L) (*p* < 0.001). Based on the diabetes-diagnosing FBG threshold values of our own (>7.9 mmol/L) and Wang’s (≥11.0 mmol/L) [21], type 2 diabetic mice were successfully generated in our experiment by feeding HFD. The FBG level in the diabetic model control group (denoted as M in Figure 1) was gradually increased and reached up to 20 mmol/L by the end of the experiment, indicating that the diabetes of model mice without any medication was aggravated with the continuous feeding of HFD. Compared to the model group, all the four medications could effectively inhibit the further increase of HFD-induced FBG levels. Meantime, they all decreased FBG levels of medicated diabetic mice greatly during the experiment excluding at Week 3. However, metformin possessed the better capability to control and decline FBG of diabetic mice than all three Chinese herbal medications (RG, CC, and RG/CC), which was observed by Waisundara et al. [15], Lin et al. [17] and Wang et al. [21]. During 56 days’ experiment, MET-treated group had the relatively stable FBG, ranging from 5.40 ± 0.74 mmol/L (at Week 0) to 9.37 ± 0.92 mmol/L (at Week 3). Some individuals administered with RG and CC or RG/CC showed the varying FBG levels during the experiment, for example, at Week 4, 6 and 7. There was no significant difference in declining FBG level of diabetic mice among RG, CC, and RG/CC groups, which was also observed by Wang et al. [21].

### 2.2. RG, CC, and Their Combination Improved Glucose Tolerance of Diabetic Mice

**Figure 2:**
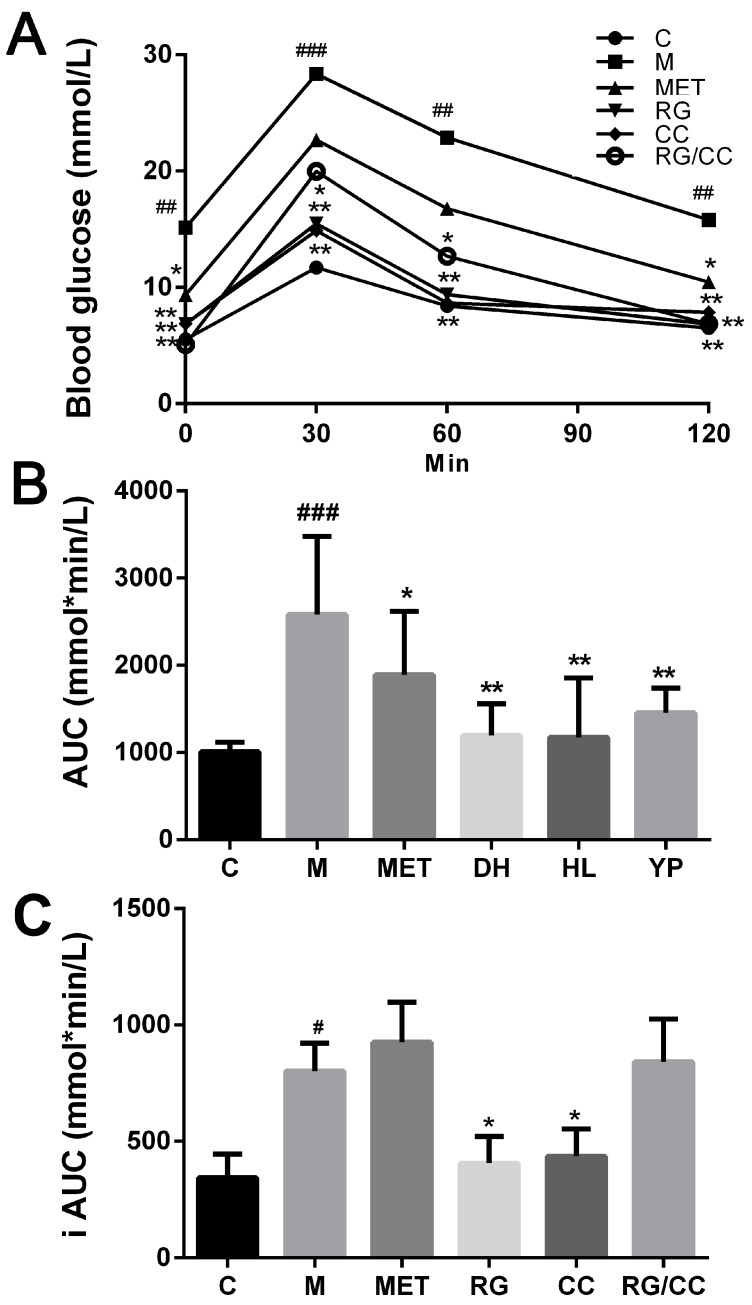
Results of a glucose tolerance test in diabetic KK-Ay mice performed at the end of 56 days of treatment. (**A**) blood glucose level after glucose loading; (**B**) AUC: area under the curve of OGTT; (**C**) iAUC: incremental area under the curve of OGTT. ^#^ *p* < 0.05, ^##^ *p* < 0.01 and ^###^ *p* < 0.001 meant significant difference of the measurement in the indicated group compared with the normal control group (C); * *p* < 0.05 and ** *p* < 0.01 meant significant difference of the measurement in the indicated group compared with the model control group (M).

On the 8th week of treatments, an oral glucose tolerance test (OGTT) was conducted to evaluate whether administration of RG and CC and RG/CC improved the antihyperglycemic activities of HFD-fed diabetic mice. After glucose administration, the blood glucose levels in mice of all the groups peaked at 30 min and then decreased nearly to their initial levels two hours later (Figure 2A). The model group showed a poorer glucose tolerance, compared with the control mice and the medicated groups. The levels of blood glucose in all four medicated groups and the control group were significantly lower than those of the untreated diabetic KK-Ay mice at each corresponding time point (*p* < 0.05, *p* < 0.01). Compared with RG/CC- and MET-administered groups, the glucose levels of RG- and CC-administered mice showed a faster reduction and restored close to the control status one hour after OGTT. The MET-administered group had a significantly lower glucose concentration than the model group until two hours after OGTT. It was observed that the area under the curve (AUC) in the model group (2582.33 ± 895.79 mmol*min/L) was significantly higher than that in the control group (1007.50 ± 111.81 mmol*min/L) (Figure 2B). The AUCs in RG-, CC- and RG/CC- administered groups were markedly decreased compared with the model mice. The results indicated that all three herbal recipes had the ability to enhance the glucose tolerance of diabetic mice. However, the incremental AUC value was found to be significantly lower only in RG and CC groups than the model mice (Figure 2C). RG/CC had the higher incremental AUC due to the quickly increased glucose at 30 min than the single herbal treatment groups. 

### 2.3. RG, CC, and Their Combination Similarly Modulated Serum Lipid Levels of Diabetic Mice 

Dyslipidemia commonly occurs in diabetic organisms. Effects of RG, CC, and their combination on serum lipid metabolism of diabetic mice were assessed by the lipid indicators including TG, TC, HDL-C and LDH-C (Table 1). Compared with the control mice, the levels of serum TG, TC and LDL-C in untreated diabetic mice were significantly increased by feeding HFD whereas the serum HDL-C was significantly reduced. The elevated levels of serum TG, TC and LDL-C in HFD-induced diabetic mice were significantly decreased by administration of metformin, RG, CC, and RC/CC (*p* < 0.05 or *p* < 0.001). The serum HDL-C level in diabetic mice was significantly elevated by administration of RG decoction (*p* < 0.01). Compared with the diabetic model mice, administration of RG/CC showed the greater reduction of serum TC, TG and LDL-C by 31.89%, 36.87% and 42.22%, respectively. As reported by Zao et al. [20], no significant difference in modulating the lipid metabolism in diabetic mice was observed among these three herbal recipes, according to our four analyzed lipid indicators.

### 2.4. Effects of RG, CC, and Their Combination on Serum Metabolomics of Diabetic Mice

#### 2.4.1. UPLC-Q/TOF-MS Method Validation

**Figure 3:**
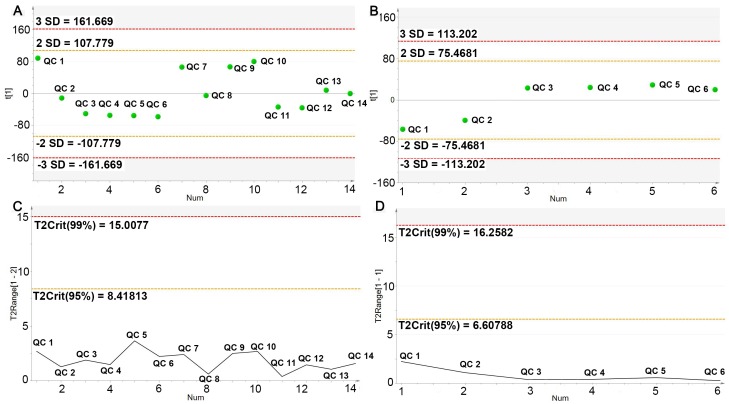
PCA line score plots of different injections of quality control (QC) sample. X-axis represented the run order of QC sample; Y-axis represented standard deviation (**A**,**B**) and Hotelling’s T2 range (**C**,**D**), separately. (**A**,**C**) for ESI^+^ mode; (**B**,**D**) for ESI^−^ mode.

The serum samples were analyzed within 15 min by UPLC-Q/TOF-MS in both positive and negative ESI detection modes. The representative chromatograms were shown in Figure S1. To guarantee the significant differences of serum metabolites in LC-MS originating from the inherent differences between groups rather than from the instrumental drift, the instrument stability and analytical repeatability were evaluated by analyzing quality control (QC) samples during the analytical run. The instrument repeatability and method repeatability were validated by analyzing one QC sample in six continuous times and six replicates of QC samples, separately. Five ion peaks (274.2753_4.33, 520.3415_5.85, 496.3415_6.29, 522.3569_6.55 and 524.3724_7.52) from the positive ion mode were extracted for method validation. For instrument repeatability, relative standard deviation (RSD)% of the peak intensities and retention times were estimated to be 1.71–11.78% and 0–0.12%, respectively; for the method repeatability, their RSD% values were separately 2.18–13.15% and 0–0.13%. Meanwhile, the deviation variation of all QC samples was further accessed via principal component analysis for method validation. The results showed that 14 QC samples in the positive ion mode and six in negative ion mode both fell within the 2 SD’s region and 95% confidence interval (Figure 3). QC samples were also further subjected to principal component analysis (PCA) and OPLS-DA with the experimental samples (Figure S2). The score plots showed that most QC samples were clustered closely. These data indicated that the analytical platform provided an excellent precision and repeatability required for a large-scale metabolomics study.

#### 2.4.2. Global Multivariate Analysis of Serum Metabolomics 

**Figure 4:**
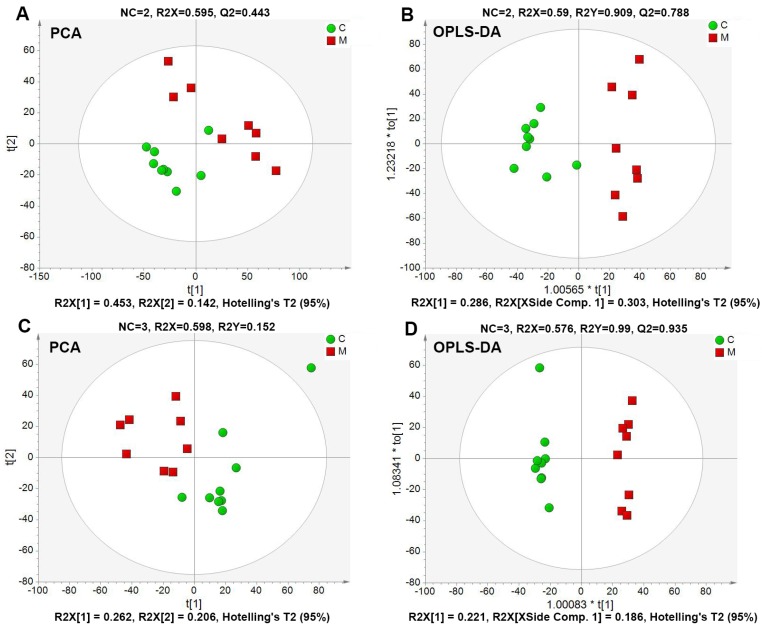
PCA and OPLS-DA score plots derived from UPLC-Q/TOF-MS profiling of the sera metabolomics of control and model groups. ESI^+^ mode: (**A**,**B**); ESI^−^ mode: (**C**,**D**); NC represented the number of components. R2X and R2Y were cumulative variation of all R2Xs and R2Ys, separately. Q2 was the cumulative predicted fraction. *p*_CV-ANOVA_ values for OPLS-DA models (**B**,**D**) were 5.20008 × 10^−4^ and 2.14075 × 10^−5^, respectively.

According to the metabolic profiling of mouse serum samples, 3299 positive ion peaks and 786 negative ion peaks were detected, respectively. Two main multivariate statistical methods were used: the unsupervised PCA to observe the general clustering trends of experimental samples, and the supervised orthogonal partial least squares discriminant analysis (OPLS-DA) to construct the classification models and distinguish the differential variables between the compared groups. In our study, the clear separation between the model and control groups was observed in both PCA and OPLS-DA score plots, indicating that the metabolic profiles differed significantly among them (Figure 4). R2 and Q2 are the parameters assessing the fitness and prediction capabilities of the established model, respectively. In the positive ion mode, the PCA produced two principal components, and the cumulative R2 and Q2 were 0.595 and 0.443. OPLS-DA resulted in two predictive components with R2X (cum) = 0.590, R2Y (cum) = 0.909, Q2 (cum) = 0.788. In the negative ion mode, the R2 and Q2 parameters of the PCA model with three principal components were 0.598 and 0.152, respectively. OPLS-DA obtained one predictive component and two orthogonal components with R2X (cum) = 0.576, R2Y (cum) = 0.990, Q2 (cum) = 0.935. The predictive capacities of Q2 (cum) in two ion modes were 78.8% and 93.5%, separately, indicating the robust fitness and predictive ability of two constructed OPLS-DA models. Meanwhile, the coefficient variability analysis of variance (CV-ANOVA) was further used to validate OPLS-DA models. The *p* values of CV-ANOVA in two established models were 5.20008 × 10^−4^ and 2.14075× 10^−5^, respectively. This result suggested that there were differential metabolites in sera between normal and diabetic mice.

**Figure 5:**
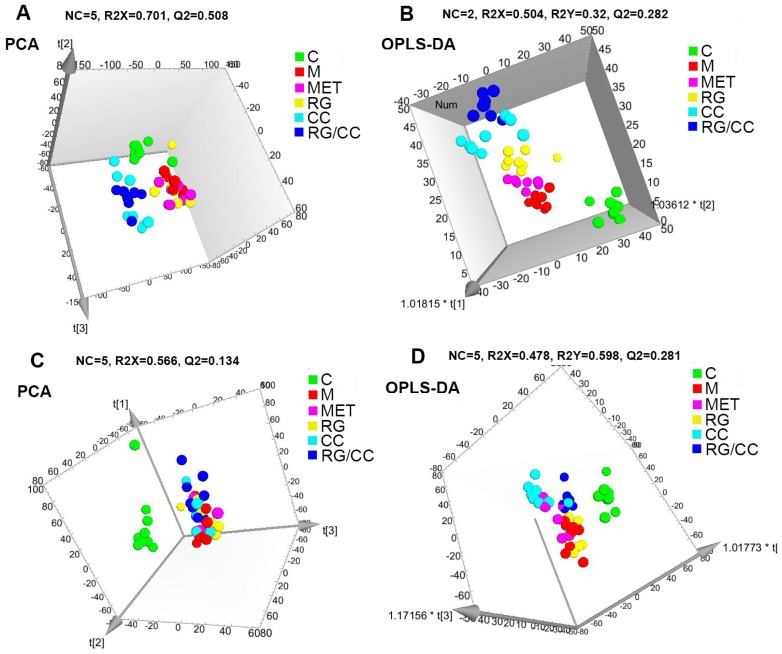
Different groups of serum metabolites visualized using PCA and OPLS-DA score plots. ESI^+^ mode: (**A**,**B**); ESI^−^ mode: (**C**,**D**). NC represented the number of components. R2X and R2Y were cumulative modelled variation of all R2Xs and R2Ys, separately, and Q2 was the cumulative predicted fraction. *p*_CV-ANOVA_ values for OPLS-DA models (**B**,**D**) were 4.42718 × 10^−8^ and 0.0105384, respectively.

Our above biochemical results showed that administration of RG and CC and RG/CC could modulate the increased glucose and abnormal lipid metabolism in serum of diabetic mice. To reveal the hypoglycemic effects of administration of RG, CC, and their combination on diabetes at the metabolite-wide level, serum samples from five different treatments of mice were collected at the end of administration for UPLC-Q-TOF-MS metabolomic analysis. The obtained serum metabolomic data were further subjected to PCA and OPLS-DA analyses (Figure 5). In the positive ion mode (Figure 5A,B), CC- and RG/CC-treated diabetic groups were clearly separated from the control and model groups; RG- and MET-treated diabetic groups were closer to the model group. This observation indicated that RG, CC, and their combination altered the metabolite profiling in diabetic sera. CC- and RG/CC-treated groups were clustered closer than RG administered group, indicating that CC and RG/CC had more similar pharmacological effects on serum metabolome of diabetes, compared to RG. The OPLS-DA result produced two predictive components and the cumulative of R2 and Q2 were R2X (cum) = 0.504, R2Y (cum) = 0.320, Q2 (cum) = 0.282. In the negative ion mode (Figure 5C,D), the PCA and OPLS-DA score plots showed an obvious separation of the control group from the model group and medication groups. Although PCA showed that the medicated groups were not well separated from the model diabetic group, the medicated groups especially CC- and RG/CC-treated diabetic groups trended to deviate from the model group (Figure 5C). This separation was more obvious in OPLS-DA model (Figure 5D). The autofitting result of OPLS-DA generated five predictive components with R2X (cum) = 0.478, R2Y (cum) = 0.598, Q2 (cum) = 0.281. Similarly, as revealed in positive ion model, CC- and RG/CC-treated groups were clustered closer than RG- administered group.

#### 2.4.3. Modulation of RG, CC, and Their Combination on HFD-Induced Diabetic Biomarkers

**Figure 6:**
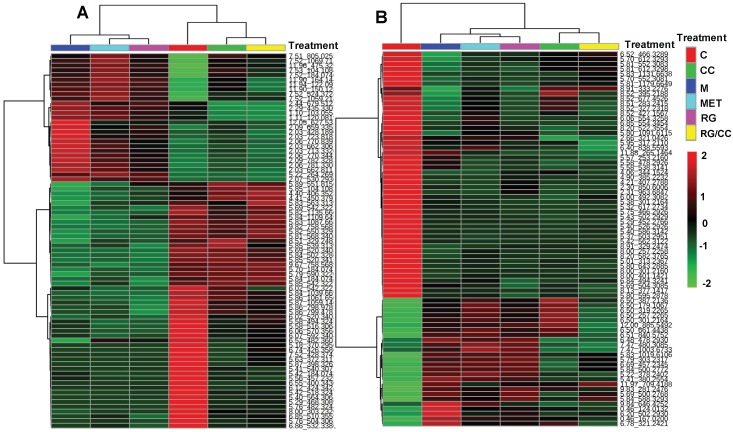
Hierarchical clustering analysis of HFD-induced differential metabolites among different treatments. The heatmaps were generated in MetaboAnalyst 4.0 (http://www.metaboanalyst.ca/) using the differential biomarkers of importance identified between the normal control group (C) and diabetic model control group (M). (**A**) ESI^+^ mode; (**B**) ESI^−^ mode. The results showed that CC and its combination with RG were clustered closer to the undiabetic control group, indicating that CC served as a sovereign drug and had a better modulation on diabetes-related metabolites in serum than RG alone and the positive drug metformin.

**Figure 7:**
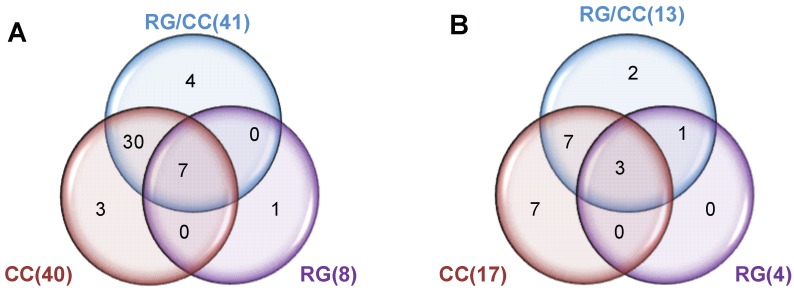
Venn diagrams showing unique or overlapped modulation of RG, CC, and RG/CC on HFD-induced biomarkers in sera of diabetic mice. The number in () was the modulated variables of the herbal medication on HFD-induced biomarkers compared to the model group. The numerical values in the diagrams depicted the metabolites that are unique to or shared between three medications. (**A**) ESI^+^ mode; (**B**) ESI^−^ mode.

Figure 6 revealed the medication regulation of the HFD-induced metabolites in diabetes. In the positive ion mode, a total of 79 metabolites were found express differently in diabetic model group compared to the control mice. 25 of these differential metabolites increased and 54 decreased in diabetic model mice (Figure 6A). In the negative ion mode, a total of 76 metabolites showed the differential expression in diabetic model group from the control mice. 26 of these differential metabolites were elevated by HFD in diabetes and 50 were reduced, compared with the control mice fed the normal diet (Figure 6B). Heatmap clustering on these diabetes-regulated metabolites showed that MET- and RG- administered mice had the similar metabolite profiles as the diabetes model group (M) while RC- and RG/CC- administered groups were closer to the normal control group (C) (Figure 6A,B), indicating that CC and RG/CC showed the better modulating capacity on metabolite changes in diabetes. Among HFD-induced variables, five ESI^+^ and three ESI^−^ variables decreased by MET administration, compared to the diabetic model group. In the positive mode, three herbal treatments totally reversed the concentrations of 45 of 79 HFD-induced ESI^+^ variables, comprised of 20 increased/25 decreased. In the negative mode, three herbal treatments changed the abundance of a total of 20 variables among 76 HFD-induced ESI^−^ biomarkers including 13 increased and 7 decreased. Venn diagrams showed that 40/17 and 41/13 were significantly reversed by CC and RG/CC in sera of diabetic mice, while 8/4 were regulated by RG administration (Figure 7A,B). Seven ESI^+^ and three ESI^−^ biomarkers were commonly regulated by three herbal medications; 3/7, 1/0 and 4/2 (ESI^+^/ESI^−^) variables were uniquely modulated by CC, RG, and RG/CC, separately. A total of 15 diabetes-related biomarkers with the supported fragment ions (Table S1) were finally annotated and mapped mainly onto lipid metabolism (Table 2). These annotated biomarkers were mainly modulated by CC and its combination with RG, which demonstrated the major function of CC in diabetes treatment at the metabolite level.

#### 2.4.4. RG and CC Showed the Same Restoring Trends on Metabolites in Diabetic Mice

**Figure 8:**
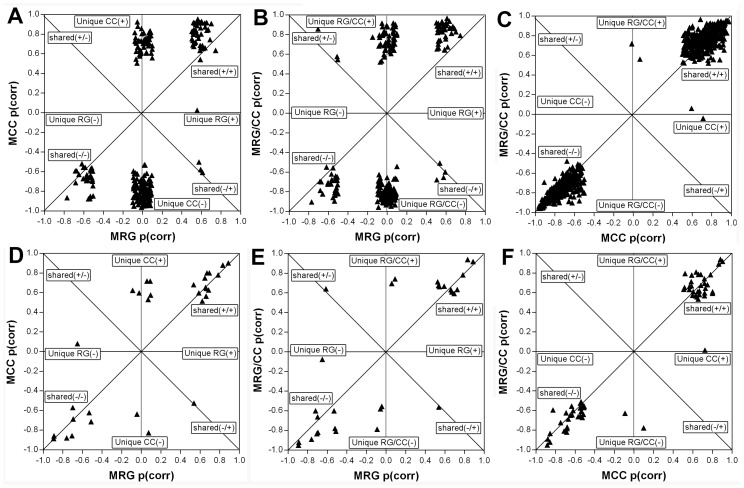
SUS-plots showing the shared and unique metabolites between herbal treatments. (**A**–**C**): based on OPLS-DA models under ESI^+^ mode. (**D**–**F**): based on OPLS-DA models under ESI^−^ mode. MRG: Model vs. RG; MCC: Model vs. CC; MRG/CC: Model vs. RG/CC. The displayed metabolites were selected based on the |p(corr)| ≥ 0.5 and the jack-knifed confidence interval and showed the significant difference (adjusted *p* < 0.5) between at least one compared treatment and the model control group. To identify the herbal treatment-unique metabolites, the |p(corr)| threshold of such metabolites was ≤0.1 in its opposite OPLS-DA model.

To find the trends of medication-responsive metabolites among three herbal treatments, we applied SUS-plot analyses on the OPLS-DA models of MCC and MRG, MRG/CC and MRG, and MRG/CC and MCC (Figure 8A–F). Although less unique metabolites in diabetes were altered by RG medication than CC (Figure 8A,D), RG and CC had the same restoring trends on metabolite levels in diabetes (Figure 8A–F), which provided the metabolomic support that they both could be used as the antidiabetic herbal medicine.

## 3. Discussion

DM is mainly characterized by hyperglycemia and dyslipidemia accompanied with biochemical alterations of lipid metabolism. The control of blood glucose level close to normoglycemia in diabetic patients is one of major therapeutic targets to reduce or slow down diabetes risks since hyperglycemia leads to many fatal organ dysfunctions and complications including retinopathy, nephropathy, neuropathy, and cardiovascular disease. *R. glutinosa* and *C. chinensis* are two of most frequently prescribed herbs in treatment of DM in ancient and modern TCM formulae [30,31,32]. Dried root of *R. glutinosa* (Sheng dihuang in Chinese) had the better antidiabetic effects than its prepared product which was steamed with wine (Shu dihuang in Chinese) [28,33,34], whereas the better antidiabetic effects were obtained by the processed radix of *C. chinesis* such as stir-fried with *Evodia rutaecarpa* decoction or wine and steamed with wine than its dried raw radix [35]. In the clinic practice, it was suggested that a high dose of *C. chinesis* decoction (≥15 g) should be taken in a short time (1–3 months) to remarkably decrease the elevated blood glucose level of diabetic patients at the early diabetic stage while a low dose of *C. chinesis* pill or powder was prescribed during the long-term therapy to maintain the lowered blood glucose level of diabetic patients [36]. A higher amount of *R. glutinosa* (6 g·kg^−1^) had the stronger hypoglycemic and hypolipidemic effects in HFD-STZ-induced diabetic mice, compared to the doses 4 g·kg^−1^ and 2 g·kg^−1^ [33]. It was reported that 10 g (kg·day)^−1^ dose of RG and CC and their combinations could effectively decrease blood glucose and lipid levels of diabetic mice into the normal range [19]. In addition, the combined CC and RG at the ratio of 1:1 showed the better blood lipid indicators (TC, TG, HDL and LDL) than the CC/RG at the ratio of 1:8. Based on these previous results, high doses of decoctions from unprocessed RG radix, processed CC radix and their formulae at the ratio of 1:1 were administered to spontaneous diabetic KK-Ay mice and their antidiabetic effects were compared. Our results demonstrated that RG, CC, and their combination RG/CC all could significantly suppress the continuous increases of HFD-induced FBG level and lower it into a close normal level especially in the early stage of diabetic mice. However, their FBG control capacities were poorer than metformin and showed more variations over eight weeks of medication administration, which reflected that it was difficulty in maintenance of diabetes therapeutic effects just depending on a long-term herbal medication. Although all three herb medications ameliorated glucose tolerance in diabetic mice; however, the single prescription RG and CC declined the glucose level faster within a short time than their combined prescription RG/CC and metformin. 

Our UPLC-Q/TOF-MS-based serum metabolomic analysis revealed that administration of RG and CC, RG/CC could reverse a total of about 30% (ESI^−^)–50% (ESI^+^) of diabetes-induced metabolite changes. Although RG and CC and their combination showed no significant difference in amelioration of FBG and serum lipid levels in HFD-induced diabetes, our metabolomic analysis revealed that they showed the differential restoring abilities in diabetes-induced metabolite disturbance which were mainly annotated as lipid compounds. CC and RG/CC showed the better modulating capacity on metabolite changes in diabetes, which was consistent with urine metabolomic comparisons of diabetic mice administered RG, CC, and RG/CC [19]. The antidiabetic difference of RG and CC at the metabolite levels may indicate that these two herbs had different action mechanisms in type 2 diabetes mellitus (T2DM) possibly due to their different antidiabetic bioactive compounds [37,38,39,40,41,42,43,44]. Therefore, it is interesting to further investigate and compare metabolomic changes caused by these compounds such as catalpol from RG radix and berberine from CC rhizoma. In addition, in the viewpoint of TCM, diabetes is caused by “yin deficiency and dryness-heat” of the body and is divided into four syndrome stages: stagnancy, heat, deficiency and damage [45]. Heat symptom occurred in the early or middle stage of DM and led to Qi and yin deficiency with the progress of DM. Although RG and CC are both heat-clearing cold drugs, CC was thought as a monarch drug and effective mainly on heat syndrome which triggered the early pathogenesis of T2DM, while raw RG nourished yin deficiency syndrome accompanied with the progress of T2DM [19,46]. Different TCM diabetic syndromes showed differential metabolites [46,47,48] and require syndrome-differentiated dependent medications [45]. In terms of TCM, excessive intake of fat often produces the unendurable heat of the body to trigger diabetes. In this experiment, we fed KK-Ay mice HFD for 56 days to induce diabetic symptoms such as hyperglycemia and hyperlipidemia. It was possible that no obvious yin deficiency was created in mice model within our experimental duration. However, these differential metabolomic effects of CC and RG observed in our experiment need to be further investigated in clinical patients with obvious different DM syndromes. 

In summary, administration of RG and CC alone and their combination could decrease the FBG level, ameliorate the tolerance of glucose, and recover the TC, TG, HDL-C and LDL-C levels to various degrees in sera of diabetic mice. A UPLC-Q/TOF-MS-based untargeted metabolomics approach combined with the multivariate data analysis was carried out to further reveal their antidiabetic effects of RG and CC alone and their combination at the metabolite level, which functioned mainly on lipid metabolism. The metabolomic difference among RG and CC and RG/CC revealed that they showed the differential restoring abilities in diabetes-induced metabolite disturbance, which provided a scientific support for their different roles in diabetic treatment. Our further SUS-plot analysis revealed that there were less unique metabolites regulated by RG.

## 4. Materials and Methods 

### 4.1. Chemicals and Reagents

HPLC-grade methanol and acetonitrile were purchased from the Fisher Corporation (Fair Lawn, NJ, USA), and formic acid (HPLC grade) was supplied by the Tianjin Reagent Company (Tianjin, China). Distilled water was obtained from Wahaha Group Co., Ltd. (Hangzhou, China). The positive control drug metformin (98% of purity) was obtained from Bristol-Myers Squibb Co., Ltd. (Shanghai, China). The assay kits for total cholesterol (TC), triglyceride (TG), high-density lipoprotein cholesterol (HDL-C) and low-density lipoprotein cholesterol (LDL-C) were purchased from BioSino Bio-Technology & Science Inc. (Beijing, China).

### 4.2. Preparation of RG, CC, and RG/CC Decoction

Fresh tuberous roots of RG var. Wen 85-5 were collected from the experimental field at Institute of Medicinal Plant Development (IMPLAD), Beijing, China and dried in the oven. The dried processed roots of CC grown in Sichuan province were purchased from Beijing Tongrentang Co., Ltd. (Beijing, China). The equal amount of root powders of RG and CC, RG/CC (RG:CC, 1:1) were weighed and immersed separately in 10× volumes of distilled water for one hour, and then boiled for 30 min. The extracted solution was filtered, and the residue was reextracted once in the same way. Finally, these two filtrates were combined, concentrated to 1 g/mL at 50 °C under the depressurized condition. The decoctions were stored at 4 °C for the subsequent administration experiment. 

### 4.3. Animals and Treatments

The animal experiments were performed in strict accordance with the Guidelines of National Health Institutes of China for the Care and Use of Laboratory Animals and approved by the Institutional Animal Care and Welfare Committee of IMPLAD with the certificate No. SYXK2013-0023 (Jing). 50 eight-week-old male KK-Ay mice with the weight of 33–36 g and eight age-matched male C57BL/6J mice with the weight of 21–23 g were purchased from Beijing HFK Bioscience Co. Ltd., China (No. SCXK (Jing) 2014-0004). All the mice were housed at the temperature of 22–23 °C and a relative humidity of 55 ± 5%, and in a 12 h light/dark cycle at the animal center of IMPLAD, Beijing, China. C57BL/6J mice were fed with regular diets and KK-Ay mice with HFD (KK Diet 1K65), which were both obtained from Beijing HFK Bioscience Co. Ltd., China. In the first two weeks after arrival, FBG levels for each KK-Ay and C57BL/6 J mouse were measured weekly using blood glucose testing strips (ACCU-CHEK Active, Roche Diagnostics GmbH, Mannheim, Germany). The KK-Ay mice with both FBG values >7.9 mmol /L were used as diabetic models in the latter administration experiments and randomly divided into five groups containing 7 to 10 mice each. Four groups of diabetic KK-Ay mice were dosed daily by gavage with MET (200 mg/kg), RG, CC, and RG/CC (10 g/kg each) [29], respectively. Simultaneously, another fifth KK-Ay mice group (diabetic model control, abbreviated as M) and C57BL/6 J mice (normal control, abbreviated as C) were given just with the same volume of normal saline. During eight weeks of administration experiment, individual body weight was recorded weekly and the dose for each treatment was adjusted to 10 g/kg/day or 200 mg/kg/day, according to the changed body weight. 

### 4.4. Evaluation of Antidiabetic Activities of RG, CC, and Their Combination

#### 4.4.1. Determination of Fast Blood Glucose and Glucose Tolerance

The tail FBG concentrations of control and administered mice were measured at the regular time weekly for eight weeks using blood glucose testing strips (ACCU-CHEK^®^ Active, Roche Diagnostics GmbH, Mannheim, Germany).

On the final day of the experiment, the KK-Ay mice and C57BL/6J mice were subjected to OGTT. Mice fasted for 10 h were given intragastrically with 2 g/kg glucose, and blood from the tail vein was subsequently collected to determine blood glucose levels at 0 (before glucose injection), 30, 60 and 120 min after glucose loading. The AUC for blood glucose concentration of each treatment was further calculated in GraphPad Prism 6.0 (La Jolla, CA, USA) using the trapezoidal formula: 0.5 × (A_0_ + B_30_)/2 + 0.5 × (B_30_ + C_60_)/2 + (C_60_ + D_120_)/2, where A_0_, B_30_, C_60_, and D_120_ referred to the glucose concentrations measured at the indicated times (subscript numbers) during OGTT. The iAUC was calculated by all area below the curve and above the fasting concentration, with any area beneath the fasting level being ignored.

#### 4.4.2. Biochemical Assays

Mice were sacrificed 12 h after OGTT. The eyeballs were removed to collect blood samples. Serum samples were separated via centrifugation at 3500 r/min at 4 °C for 10 min and each sample was divided into two parts. One part was used for biochemical analysis, and the other was preserved at −80 °C for further UPLC-Q/TOF-MS analysis.

The concentrations of serum TC, TG, HDL-C and LDL-C were determined using auto-biochemical analyzer (XL-640, Erba, Germany) following the protocols of commercial enzymatic kits.

### 4.5. Serum Metabolomic Analysis

#### 4.5.1. Preparation of Serum Samples and QC Samples

All frozen serum samples were thawed and equilibrated at 4 °C prior to analysis. 200 μL serum was mixed with 600 μL precooled (4 °C) acetonitrile (ACN) and vortexed for 30 s. The mixture was incubated on ice for 15 min and then centrifuged at 15,000 rpm at 4 °C for 15 min. The supernatant was collected and filtered via a 0.22 μm microporous membrane, and then transferred into an auto-sampler vial for the subsequent UPLC-Q/TOF-MS analysis. The QC sample was mixed with 10 μL aliquot of all the samples and prepared in the same way as the aforementioned serum samples. QC sample was injected recurrently throughout the analytical run to monitor the LC/MS system.

#### 4.5.2. UPLC-Q/TOF-MS Analysis

The serum samples were separated at 40℃ on a Waters ACQUITYTM UPLC system (Waters Corporation, Milford, MA, USA) equipped with a Waters UPLC BEH C18 column (2.1 mm × 100 mm, 1.7 μm). The mobile phase included a binary solvent system with water containing 0.1% formic acid (A) and ACN (B), and run under the following optimized gradient program: 0–0.5 min, 5% B; 0.5–2 min, 5–30% B; 2–5 min, 30–60% B; 5–8 min, 60–80% B; 8–11 min, 80–100% B; 11–13 min, 100–5% B; 13–15 min, 5% B. The injection volume was 5 μL and the flow rate was set to 0.2 mL/min. Six consecutive injections of QC sample were initially done in the positive mode to stabilize the analytical platform and used for repeatability of instrument. Experimental samples were injected in the group sequence: normal group, model group, MET group, RG group, CC group and RG/CC group. In addition, one QC sample was inserted between two neighboring groups. 

The Q-TOF-MS analysis was performed in both positive and negative ion modes on a Synapt G2 high definition mass spectrometer system (Waters Corporation, Milford, MA, USA) fitted with an ESI ion source. The operating parameters of the MS analysis were set as: capillary voltage, 3kV for the positive ion mode and 2.8 kV for the negative ion mode; source temperature, 120 °C; cone gas flow, 50 L/h; desolvation gas temperature, 450 °C; desolvation gas flow, 600 L/h; collision energy, 6 V; cone voltage, 40 V. The TOF-MS data were acquired in centroid mode over the *m*/*z* range of 50–1200 Da with a scan rate of 0.15 s and an inter-scan delay of 0.02 s.

#### 4.5.3. Data Processing and Multivariate Analysis

The original MS data were imported to MarkerLynx Applications Manager package of MassLynx software (version 4.1, Waters Corp., Manchester, UK) for feature detection, retention time alignment and normalization to total ion intensity. Detailed parameter settings of peak extraction and alignment were set as follows: peak width at 5% height, 1 s; peak-to-peak baseline noise, 0.05; no smoothing; noise elimination level, 6; Deisotope data; marker intensity threshold, 300; mass tolerance, 0.05 Da; mass window, 0.05 Da; RT window, 0.2 min. The resulting dataset including RT-*m*/*z* pairs, sample names, and corresponding normalized ion intensities were acquired and then fed into SIMCA-P software (V.13.0, Umetric, Umea, Sweden) for the multivariate analysis (MVA). Prior to MVA, each peak was mean-centered and scaled to Pareto variance. After data preprocessing, unsupervised PCA and orthogonal to partial least squares-discriminate analysis (OPLS-DA) were applied to observe the global clustering trends of various groups and visualize their distribution. The cumulative R2 and Q2 were calculated to evaluate the fitness and predictive capability of two constructed pattern recognition models. *p*_CV-ANOVA_ values were calculated to assess the quality of OPLS-DA models. 

The scores of the variable importance in projection (VIP) reflect the contribution of the analyzed variables to OPLS-DA model. Variables that were differentially expressed between the normal control group and diabetic model group were selected according to VIP > 1, |P(corr)| value ≥ 0.65 and Jack-knifed confidence intervals (CIJFjk) excluding 0. Furthermore, one-way analysis of variance (ANOVA) with Newman-Keuls multiple comparisons test was performed in GraphPad Prism 6.0 (La Jolla, CA, USA) to determine the statistical significance of the HFD-induced diabetic biomarkers between two compared groups including the medication groups. The shared and unique metabolites to herbal treatments were obtained via SUS-plot analysis in SIMCA-P software, following the strategies [49]. Identification of these biomarkers was done by comparison of mass-to-charge ratio (*m*/*z*) and MS/MS fragment ions with Human Metabolome Database (http://www.hmdb.ca/) and relevant published literatures. The relevant metabolic pathways of potential biomarkers were determined based on KEGG database (http://www.genome.jp/kegg/). To exhibit the variation trends of these identified biomarkers among drug treated groups, model group and normal control group, their concentrations in serum were expressed as ion intensity and reported as the means ± SD.

### 4.6. Statistical Analysis

All the results were expressed as means ± SD. The significant difference of FBG levels, serum lipid levels and AUCs obtained in OGTT between the pairwise compared groups was evaluated by Newman-Keuls multiple comparisons test in GraphPad Prism 6.0 (La Jolla, CA, USA). The adjusted *p* < 0.05 was considered statistically significant.

## Figures and Tables

**Table 1:** Effects of different treatments on serum lipids of diabetic mice after eight weeks of administration.

Treatment	TC (mmol·L^−1^)	TG (mmol·L^−1^)	HDL-C (mmol·L^−1^)	LDL-C (mmol·L^−1^)
C	2.26 ± 0.16	0.89 ± 0.16	2.88 ± 0.32	0.25 ± 0.07
M	4.17 ± 0.48 ^###^	1.98 ± 0.27 ^###^	1.91 ± 0.16 ^###^	0.90 ± 0.25 ^###^
MET	3.24 ± 0.47 ***	1.50 ± 0.35 ***	2.53 ± 0.41 **	0.58 ± 0.11 ***
RG	3.05 ± 0.45 ***	1.35 ± 0.15 ***	2.12 ± 0.35	0.59 ± 0.06 ***
CC	3.64 ± 0.24 *	0.99 ± 0.15 ***	2.49 ± 0.15 **	0.52 ± 0.10 ***
RG/CC	2.84 ± 0.41 ***	1.25 ± 0.21 ***	2.09 ± 0.30	0.52 ± 0.12 ***

^###^*p* < 0.001 Model vs. Control; * *p* < 0.05, ** *p* < 0.01 and *** *p* < 0.001 medicated mice vs. Model.

**Table 2:** Annotation of HFD-induced serum biomarkers in diabetic mice and their alteration by four medications.

VIP	RT (min)	Quasi-Molecular Ion (*m*/*z*)	Cal. *m*/*z*	Mass Error (ppm)	HMDB ID	Metabolite	Formula	Metabolic Pathway	Fold Change				
									C-M	M-MET	M-RG	M-CC	M-RG/CC
1.36	5.86	799.4781 [M + H]^+^	799.4838	−7.1339	HMDB40655	Mabioside D	C42H70O14	Lipid metabolism	2.04 ^c^	1.00	0.70	1.58 ^b^	1.35
3.59	5.69	542.3221 [M + H]^+^	542.3241	−3.6900	HMDB10397	LysoPC(20:5)	C28H48NO7P	Lipid metabolism	1.86 ^b^	1.18	1.01	1.55	1.75 ^a^
4.02	5.70	184.0747 [M + H]^+^	184.0739	4.3478	HMDB01565	Phosphorylcholine	C5H15NO4P	Phospholipid biosynthesis	1.90 ^b^	1.20	1.08	1.99 ^b^	2.02 ^b^
10.23	9.67	758.5689 [M + H]^+^	758.5694	−6.5963	HMDB08296	PC(20:1/14:1)	C42H80NO8P	Lipid metabolism	4.42 ^b^	0.11	0.72	4.67 ^a^	5.10 ^a^
15.35	5.85	520.3415 [M + H]^+^	520.3398	3.2692	HMDB10386	LysoPC(18:2)	C26H50NO7P	Lipid metabolism	1.66 ^c^	1.07	1.00	1.68 ^c^	1.57 ^b^
11.37	5.81	568.3408 [M + H]^+^	568.3398	1.7606	HMDB10404	LysoPC(22:6)	C30H50NO7P	Lipid metabolism	3.05 ^c^	1.37	1.43	2.78 ^c^	3.12 ^c^
3.28	8.52	327.2318 [M − H]^−^	327.233	−3.6697	HMDB02183	Docosahexaenoic acid	C22H32O2	Lipid metabolism	1.72 ^c^	1.28	1.10	1.41 ^a^	1.08
3.81	5.58	478.2926 [M − H]^−^	478.2939	−2.7197	HMDB11476	LysoPE(0:0/18:1)	C23H46NO7P	Fatty acid metabolism	1.71 ^c^	0.65	0.73	0.59 ^a^	0.73
2.06	6.20	502.2930 [M − H]^−^	502.2939	−1.7928	HMDB11515	LysoPE(20:3/0:0)	C25H46NO7P	Lipid metabolism	0.41 ^b^	0.61	0.69	0.53 ^a^	0.63
2.43	12.00	885.5492 [M − H]^−^	885.5499	−7.9096	HMDB09900	PI(20:4/18:0)	C47H83O13P	Lipid metabolism	0.17 ^c^	1.05	1.19	1.43	0.35 ^b^
2.07	6.78	321.2421 [M − H]^−^	321.2435	−4.3614	HMDB62747	12(S)-HETrE	C20H34O3	Lipid metabolism	0.28 ^c^	0.63 ^a^	0.66 ^a^	0.77	0.45 ^c^
1.75	5.41	380.2554 [M − H]^−^	380.2571	−4.4737	HMDB01383	Sphinganine 1-phosphate	C18H40NO5P	Lipid metabolism	0.20 ^c^	0.87	0.83	0.52 ^b^	0.55 ^a^
3.56	7.47	480.3085 [M − H]^−^	480.3096	−2.2917	HMDB10381	LysoPC(15:0)	C23H48NO7P	Lipid metabolism	0.56 ^b^	1.11	1.04	0.65 ^b^	0.55 ^b^
1.36	5.37	503.2951 [M − H]^−^	503.3014	−12.5249	HMDB33709	Desglucocoroloside	C29H44O7	Lipid metabolism	16.26 ^c^	0	0.93	0.73	0.85
1.54	11.88	265.1464 [M − H]^−^	265.1445	7.1698	HMDB35210	10-Hydroxymyoporone	C15H22O4	Lipid metabolism	1.36 ^c^	0.58 ^c^	0.43 ^c^	0.44 ^c^	0.33 ^c^

VIP (variance importance for projection) from OPLS-DA constructed with the control and model group; RT: retention time; Fold change was calculated by the ratio of the average peak intensity of the other group to model group. a, b, c represented separately the significance *p* < 0.05, *p* < 0.01 and *p* < 0.001.

## Supplementary Materials

Supplementary materials can be found at http://www.mdpi.com/1422-0067/19/12/3984/s1. Figure S1: Representative UPLC-Q/TOF-MS chromatograms of mouse serum metabolome. (A) ESI^+^ mode; (B) ESI^−^ mode. Figure S2. PCA and OPLS-DA score plots showing QC samples, Control (C), Model control (M) and medicated groups. ESI+ mode: (A, B); ESI- mode: (C, D). NC represented the number of components. Table S1: The metabolites and their fragment ions detected by UPLC-Q/TOF-MS analysis.
