# Supplementary material for: UPLC-Q/TOF-MS-Based Serum Metabolomics Reveals Hypoglycemic Effects of Rehmannia glutinosa, Coptis chinensis and Their Combination on High-Fat-Diet-Induced Diabetes in KK-Ay Mice
Source: Int J Mol Sci. 2018 Dec 11;19(12):3984. doi: 10.3390/ijms19123984 (PMC6320869; doi:10.3390/ijms19123984)
Supplement: Supplementary file 1 [file ijms-19-03984-s001.pdf]

Supplementary Material

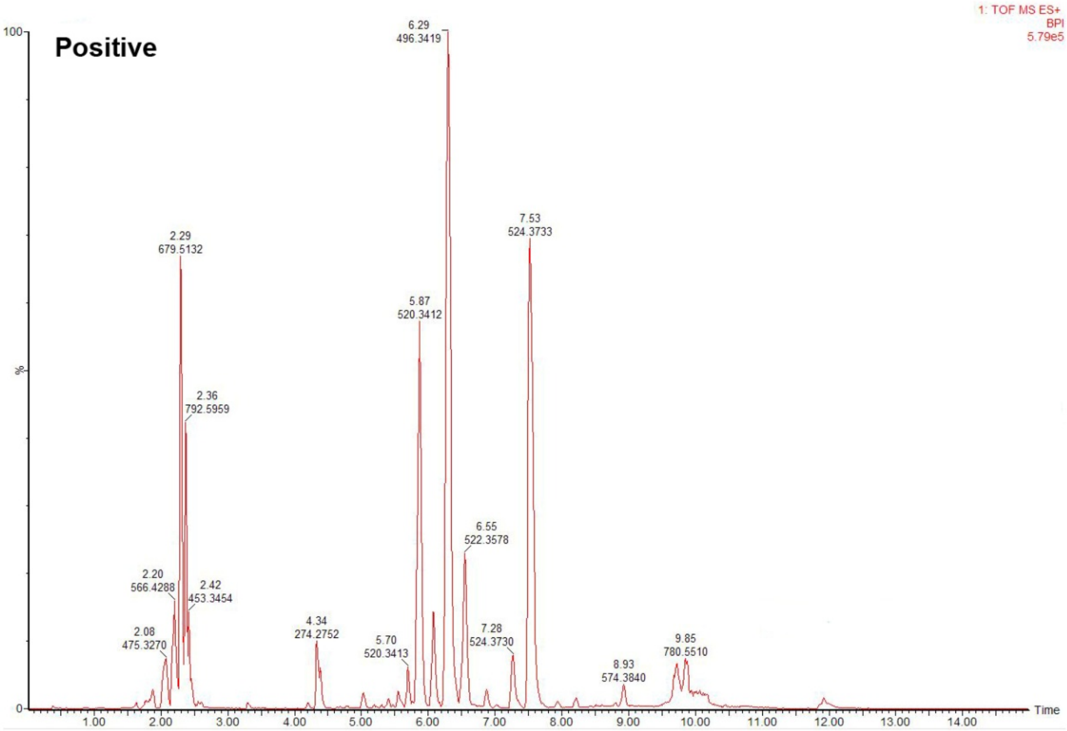

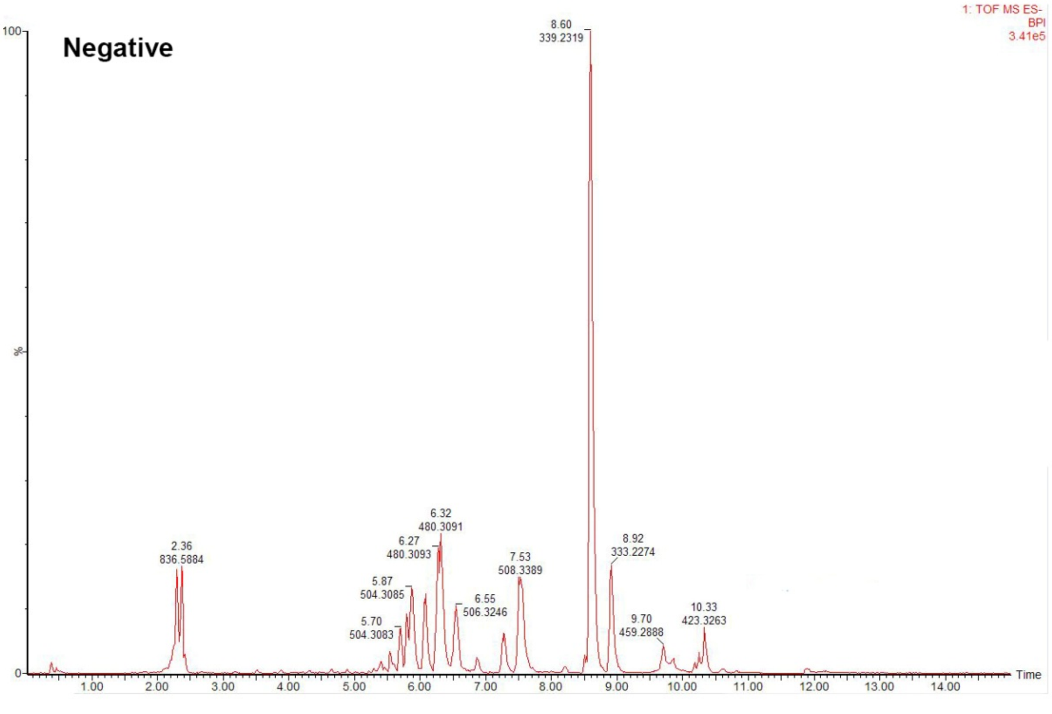

Figure S1. Representative UPLC/Q-TOF-MS chromatograms of mouse serum metabolome under positive and negative ESI modes.

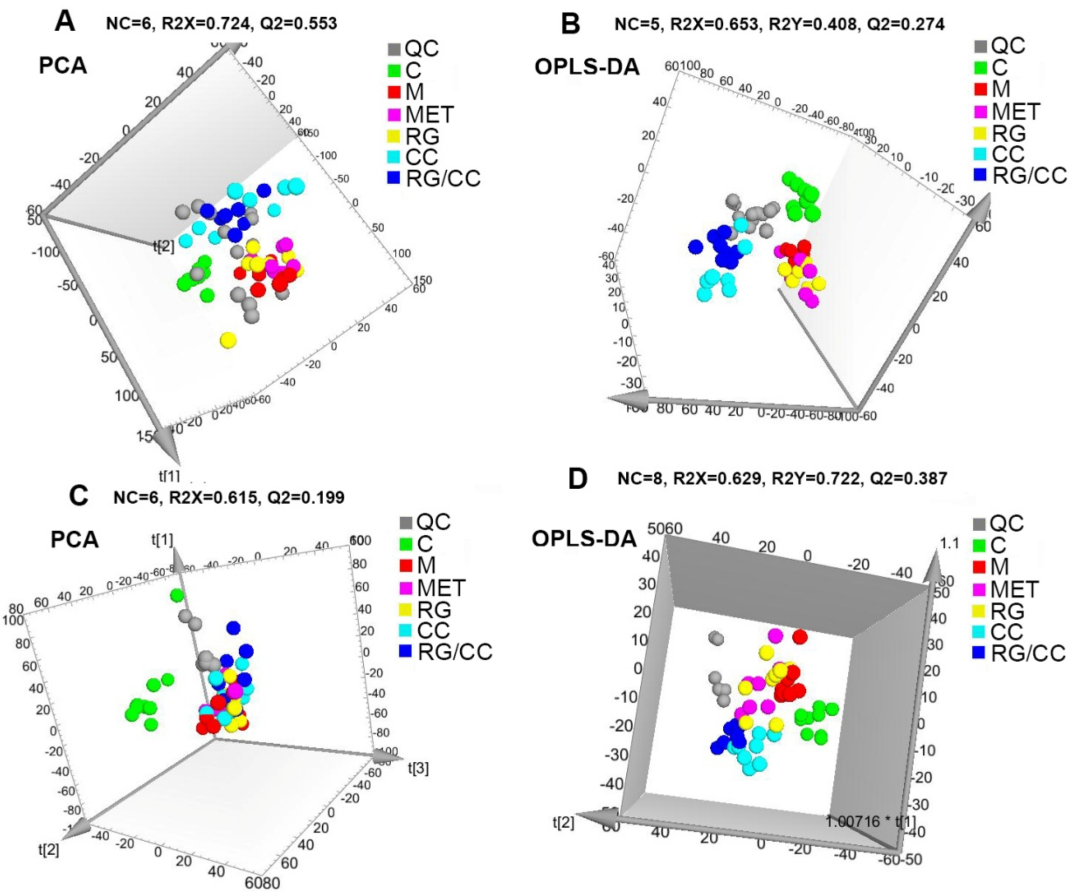

Figure S2. PCA and OPLS-DA score plots showing QC samples, Control (C), Model control (M) and medicated groups. ESI+ mode: (**A**, **B**); ESI- mode: (**C**, **D**). NC represented the number of components.

Table S1. Fragment ion information of annotated diabetes-related biomarkers.

Variable	metabolite	Formula	Fragment ion
5.86_799.4781	Mabioside D	C42H70O14	783.8951; 653.2307; 455.1884
5.69_542.3221	LysoPC(20:5)	C28H48NO7P	483.2474; 184.0738
5.70_184.0747	Phosphorylcholine	C5H15NO4P	153.1274; 125.0010
9.67_758.5689	PC(20:1/14:1)	C42H80NO8P	739.4454; 673.5170; 575.5107; 184.0753
5.85_520.3415	LysoPC(18:2)	C26H50NO7P	502.3279; 443.2553; 184.0746
5.81_568.3408	LysoPC(22:6)	C30H50NO7P	550.3302; 491.2591; 184.0743
8.52_327.2318	Docosahexaenoic acid	C22H32O2	283.2419
5.58_478.2926	LysoPE(0:0/18:1)	C23H46NO7P	419.8535; 215.0318; 197.0269
6.20_502.2929	LysoPE(20:3/0:0)	C25H46NO7P	484.8427;458.9467
12.00_885.5492	PI(20:4/18:0)	C47H83O13P	
6.78_321.2421	12(S)-HETrE	C20H34O3	285.9737; 257.8975
5.41_380.2554	Sphinganine 1-phosphate	C18H40NO5P	
7.47_480.3085	LysoPC(15:0)	C23H48NO7P	183.0100;
5.37_503.2951	Desglucocoroloside	C29H44O7	
11.89_265.1464	10-Hydroxymyoporone	C15H22O4	
